# Comparable reinfection rates between one‐stage and two‐stage revision total knee arthroplasty with gastrocnemius flap reconstruction for periprosthetic joint infection: A systematic review and meta‐analysis

**DOI:** 10.1002/jeo2.70738

**Published:** 2026-05-10

**Authors:** Jonas Sina, Daniel Schrednitzki, Andrew Ting, Daniel Addai, Bassil Azam, Priyanshu Saha, Nils Meissner, Andreas M. Halder, Michael Dietrich, Andrew James Price, Abtin Alvand, Alexander Maslaris

**Affiliations:** ^1^ Nuffield Department of Orthopaedics, Rheumatology and Musculoskeletal Sciences (NDORMS) University of Oxford Oxford UK; ^2^ Clinic for Orthopaedic Surgery, Hand Surgery and Traumatology, City Hospital Zurich Zurich Switzerland; ^3^ Department of Orthopaedic and Trauma Surgery Sana Hospital Lichtenberg Berlin Germany; ^4^ Department of Orthopaedic Surgery Sana Hospital Sommerfeld Kremmen Germany

**Keywords:** one‐stage revision, periprosthetic joint infection, revision total knee arthroplasty, soft tissue reconstruction, two‐stage revision

## Abstract

**Purpose:**

Gastrocnemius flap coverage is a widely used technique for soft‐tissue reconstruction in complex revision total knee arthroplasty (rTKA) for periprosthetic joint infection (PJI). However, clinical outcomes following one‐stage and two‐stage revision strategies in this context are poorly defined. The purpose of this meta‐analysis was to synthesize, critically appraise, systematically review and compare reinfection rates and complication profiles between one‐ and two‐stage septic rTKA for PJI using a gastrocnemius flap for reconstruction.

**Methods:**

A systematic review and meta‐analysis based on the Preferred Reporting Items for Systematic Reviews and Meta‐Analyses (PRISMA) guidelines was conducted. MEDLINE, Embase, Cochrane Library and Web of Science were searched from inception to 6 April 2025 for studies on patients undergoing rTKA for PJI with soft tissue reconstruction using a gastrocnemius flap. Outcomes of interest included reinfection rates, any complications and flap‐related complications. A pooled meta‐analysis at group level was performed to compare interventions.

**Results:**

There were 11 studies reporting on 271 rTKAs involving gastrocnemius flap reconstruction for PJI that met inclusion criteria. Of these, 56 were one‐stage rTKAs, while 215 were two‐stage rTKAs. PJI eradication rate was 66.1% in the one‐stage group versus 54.4% in the two‐stage group. There were no statistically significant differences between groups for reinfection (odds ratio [OR]: 0.61; 95% confidence interval [CI]: 0.33–1.13; *p* = 0.12), any complications (OR: 1.59; 95% CI: 0.71–3.54; *p* = 0.26) or flap‐related complications (OR: 1.03; 95% CI: 0.43–2.47; *p* = 0.94).

**Conclusion:**

It was found that one‐stage and two‐stage rTKA using a gastrocnemius flap showed comparable rates of reinfection, any complication and flap‐related complication with the data available for this meta‐analysis. Findings suggest that one‐stage revision may be a viable treatment option for suitable patients. However, higher‐quality studies are warranted to identify potential true differences within this high‐risk group.

**Level of Evidence:**

Level IV.

## Graphical abstract

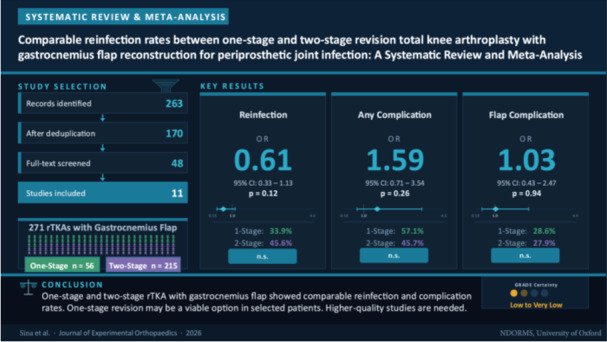

**Abbreviations**
CI: confidence Interval
FJS: Forgotten Joint Score
FU: follow‐up
GRADE: Grading of Recommendations Assessment, Development and Evaluation
KOOS: Knee Injury and Osteoarthritis Outcome Score
KSS: Knee Society Score
MDT: multidisciplinary team
NR: not reported
OKS: Oxford Knee Score
OR: odds ratio
PICO: Population, Interventions, Comparisons and Outcomes
PJI: periprosthetic joint infection
PRISMA: Preferred Reporting Items for Systematic Reviews and Meta‐Analyses
PROMs: patient‐reported outcome measures
RevMan: Review Manager
ROBINS‐I: Risk Of Bias In Non‐randomized Studies—of Interventions
ROM: range of motion
rTKA: revision total knee arthroplasty
SD: standard deviation
SF‐12: Short Form‐12
SPSS: Statistical Package for the Social Sciences
TKA: total knee arthroplasty
VAS: Visual Analogue Scale
WOMAC: Western Ontario and McMaster Universities Osteoarthritis Index

## INTRODUCTION

Primary total knee arthroplasty (TKA) is a safe and successful treatment option for end‐stage osteoarthritis [31]. Whilst rare, periprosthetic joint infection (PJI) occurs in approximately 1% of primary TKAs, a particularly devastating complication associated with an increased risk of morbidity, mortality and substantial costs [30, 33, 37].

As the number of TKA and revision total knee arthroplasty (rTKA) continues to rise, the incidence of PJI is increasing accordingly, posing a growing clinical and economic challenge [35, 37]. Re‐revision procedures are also becoming more frequent, and patients with multiple prior revisions are at particularly high risk, with infection rates exceeding those observed following primary arthroplasty [4, 10, 17].

If a revision of the TKA due to PJI is necessary, either one‐stage or two‐stage approaches are utilized. Two‐stage revision remains the most widely adopted treatment approach across centres, particularly for chronic infections, infections caused by difficult‐to‐treat pathogens and/or compromised soft tissues [6]. However, one‐stage revision is gaining in popularity because it has been shown to achieve outcomes comparable to, and in some domains superior to, those of two‐stage revision, while presenting a lower level of morbidity [3, 8, 15, 20, 46].

Revision surgery is associated with an increased risk of soft tissue damage [22]. The knee is particularly susceptible due to the scarcity of the anterior soft tissue. Each surgical procedure results in further devascularisation of the periarticular soft tissue, putting the patient at greater risk for wound complications. This is particularly problematic in the context of PJI, where soft tissue integrity is critical for infection control. Patients with PJI and concurrent soft tissue defects requiring plastic coverage are therefore at a particularly high risk of recurrent infection, implant or flap failure, amputation and death [2, 13, 44].

When there is extensive soft tissue deficiency, flap coverage is commonly performed. This aims to reconstruct a well‐vascularised soft tissue envelope conducive to infection resolution, functional preservation and eventually limb salvage. Although several techniques for soft tissue coverage have been described [1, 5, 24, 27, 32], the gastrocnemius flap remains the preferred reconstructive option for complex soft tissue defects as it has several distinct advantages. The gastrocnemius flap is anatomically versatile, supported by a reliable vascular supply and has sufficient volume for large defects. With the ability to harvest in the same surgical setting as rTKA, minimal donor site morbidity and no microvascular anastomosis required, the technique is generally a reliable option in complex rTKA [18, 42].

Whilst the use of the medial gastrocnemius flap is widely adopted for soft tissue reconstruction in rTKA, its outcomes in the context of one‐stage and two‐stage approaches for PJI management are poorly defined. Little comparative evidence is available, and to date, no comprehensive synthesis has compared the available evidence, with emphasis on reinfections and complications between these two strategies. This meta‐analysis therefore aims to synthesize, critically appraise, systematically review and compare reinfection rates, complications and clinical outcomes of patients undergoing rTKA for PJI with soft tissue reconstruction using a gastrocnemius flap, stratified by revision strategy.

It was hypothesized that one‐stage rTKA with gastrocnemius flap reconstruction is associated with reinfection and complication rates comparable to those observed after two‐stage revision in patients with PJI requiring soft tissue coverage.

## MATERIAL AND METHODS

### Registration and reporting

The study was registered at PROSPERO prior to commencement of the study (CRD420251105075) and follows the principles detailed in the Handbook of the Cochrane Collaboration, as well as the Preferred Reporting Items for Systematic Reviews and Meta‐Analyses (PRISMA) 2020 statement [29]. The completed PRISMA checklist is available in Supporting Information S1: Appendix A.

### Search strategy

The design of the search strategy and identification of databases was conducted with the assistance of an experienced information specialist from the Bodleian Libraries—University of Oxford. MEDLINE, Embase, Cochrane Library and Web of Science were searched from inception to 6 April 2025. The initial search strategy for MEDLINE, including all identified keywords and index terms, was adjusted for each included database and/or information source. There were no restrictions regarding language of publication or time period. Reference lists of included studies were screened to identify potentially relevant publications. Sources such as OpenGrey and ProQuest Dissertations & Theses were searched for potential grey literature. The full search strategy can be found in Supporting Information S1: Appendix B.

### Types of study and eligibility

Randomized and non‐randomized comparative studies comparing adults (≥18 years) undergoing rTKA for PJI with gastrocnemius flap reconstruction for soft tissue coverage were eligible for inclusion. Case reports and case series, conference abstracts, narrative reviews and studies without a clearly defined comparator group were excluded. Systematic reviews and meta‐analyses were excluded but screened for references. The full set of inclusion and exclusion criteria can be found in Table 1.

**Table 1:** Inclusion and exclusion criteria used for study selection.

Inclusion	Exclusion
Randomized controlled trials, non‐randomized controlled trials, before‐after studies, interrupted time‐series studies, prospective/retrospective cohort studies, case‐control studies and analytical cross‐sectional studies	Systematic reviews, meta‐analyses, editorials, letters, case reports, conference abstracts
Patients aged ≥18 years	Patients aged <18 years
Studies reporting on patients undergoing revision total knee arthroplasty (rTKA) for periprosthetic joint infection (PJI)	Studies reporting on rTKA for other indications (e.g., aseptic loosening, mechanical failure, malignancy)
Studies describing soft‐tissue reconstruction using a gastrocnemius flap during rTKA	Studies using other reconstruction techniques (e.g., free or rotational flaps), unless outcomes for gastrocnemius flaps are reported separately
Publications in any language	Patients with insufficient data on outcome parameters
	Studies in which the outcome parameters are not distinguishable/extractable for the target population

### Study selection

The search results were imported into Mendeley Reference Manager 1.19.8 (Elsevier) and the web application Rayyan [28]. Three independent, review authors (J. S., P. S. and D. A.) de‐duplicated and screened the abstracts according to the pre‐specified inclusion and exclusion criteria. Disagreements were resolved by discussion. The full text of each study potentially meeting the inclusion criteria was screened by the first author.

#### Population, Interventions, Comparisons and Outcomes (PICO)

##### Population

Patients aged 18 years or older with PJI of the knee requiring rTKA and soft tissue reconstruction were eligible for inclusion. Only studies in which patients received a gastrocnemius flap for soft tissue coverage for PJI were included. If the flap cohort included mixed indications, studies were only included if outcomes for patients with PJI were reported separately.

##### Intervention and Comparisons

rTKA for PJI with soft‐tissue reconstruction using a gastrocnemius flap was the primary intervention of interest. Both muscle‐only and musculocutaneous flaps were eligible. The flap procedure could be performed either as part of a one‐stage or two‐stage strategy. Studies involving flap coverage for non‐infectious indications were excluded unless outcomes of a PJI subgroup were reported separately. Comparators included one‐stage versus two‐stage revision procedures.

##### Outcomes of interest

###### PJI eradication rate

The primary outcome was PJI eradication rate, defined as the absence of clinical, microbiological or radiographic evidence of infection at follow‐up without the need for additional surgical procedures or long‐term suppressive antibiotic therapy. Data on infection eradication were extracted at 1‐year, 2‐year follow‐up when available. Studies were required to report on infection status at the final follow‐up.

###### Any complication rate

The overall rate of complication was the percentage of patients who had a minimum of one intraoperative or postoperative complication. Complications were defined as surgical, medical, wound‐related and implant‐related events. Pooled rates of complication were derived from the count of patients with a minimum of one complication out of the total count of patients per study arm. Where only the types of complication, rather than patient‐level complication overlap, were reported, aggregated complication rates were not calculated to preclude double‐counting.

###### Flap‐related complications

Any complication directly related to the gastrocnemius flap was defined as a flap‐related complication. This encompassed, but was not limited to, necrosis of the flap, either partial or total, dehiscence of the wound at the donor site or recipient site, requirement for flap revision, formation of a haematoma, delayed healing and morbidity of the donor site. All complications related to the flap procedure were selected individually, irrespective of timing. Where the distinction between flap‐related and overall surgical complications was not made, events were only counted where a direct relationship with the flap was explicitly stated or could reasonably be inferred from the text.

###### Patient‐reported outcome measures (PROMs) and functional outcomes

Instruments covering domains such as pain, function, pain and function combined, joint‐related health status or patient activity were defined as joint‐specific PROMs. These included Oxford Knee Score (OKS), the Forgotten Joint Score (FJS), the Knee Society Score (KSS), the Knee Society Score Function (KSS Function), the Knee Injury and Osteoarthritis Outcome Score (KOOS), the Short Form‐12 (SF‐12) and the Western Ontario and McMaster Universities Osteoarthritis Index (WOMAC). Other functional outcomes included range of motion (ROM) and Visual Analogue Scale (VAS) score.

### Data management and extraction

Data from all included manuscripts were extracted and collected using a standardized data extraction tool developed using Microsoft Excel (Microsoft Corporation). The data extraction form was not modified during the data extraction process. Data were extracted on study‐level characteristics including authorship, year of publication, country of origin, study design, sample size, level of evidence and duration of follow‐up. Patient‐related variables were age, sex, comorbidities, indication for revision, type of infection and history of previous revision procedures. Intervention‐specific data included surgical approach (one‐stage vs. two‐stage revision) and timing of flap placement. Functional outcomes were collected where available and included KSS, OKS, ROM, extension lag and pain scores. Information was also collected on re‐infection rates, overall complications rates and flap‐related complications.

### Statistical analysis

For dichotomous variables, odds ratios (ORs) with 95% confidence intervals (CIs) were calculated based on group‐level aggregated data. Statistical heterogeneity within one‐ and two‐stage subgroups was not assessed, as the meta‐analysis was performed on aggregated group‐level data rather than on individual study‐level effect sizes. Given the clinical heterogeneity in the studies, a random‐effects model was utilized for all pooled analyses.

### Risk of bias assessment

The risk of bias was determined using the ROBINS‐I (Risk Of Bias In Non‐randomized Studies—of Interventions) tool constructed by the Cochrane Methods Group. Seven domains for the risk of bias are assessed using the tool: bias due to confounding, participant selection, intervention classification, deviation from the intervention as planned, missing data, measurement of the outcome and selection of the result. These domains are graded as having a low, moderate, serious or critical risk of bias. Two reviewers (J. S. and P. S.) assessed the articles independently of each other. Any disagreements were resolved through discussion, involving a third reviewer (A. T.) if necessary. Ratings were used for descriptive analysis and interpretation but not as exclusion criteria.

### Certainty of evidence assessment

Certainty of the evidence was determined by using the Grading of Recommendations Assessment, Development and Evaluation (GRADE) approach. Individual Assessment for each outcome was performed with the use of the GRADEpro® tool (GRADEpro Guideline Development Tool, McMaster University, 2023). GRADE is a transparent and systematic approach commonly employed to grade evidence quality and the strength of recommendations. Risk of bias, inconsistency, indirectness, imprecision and publication bias are taken into account. According to this, the evidence certainty is estimated as high (⊕⊕⊕⊕), moderate (⊕⊕⊕◯), low (⊕⊕◯◯) or very low (⊕◯◯◯).

### Missing data

Where data were incomplete or inconclusive, study authors were contacted for further information. In five studies [7, 9, 25, 36, 44], outcome data were missing, or insufficient information was available to allow assignment. The authors were contacted and provided the relevant data and/or clarification.

### Software

Statistical analyses were performed using Review Manager (RevMan), Version 5.4 (The Cochrane Collaboration, 2020), and IBM Statistical Package for the Social Sciences (SPSS) Statistics, Version 29.0.2.0 (IBM Corporation, 2023).

## RESULTS

**Figure 1:**
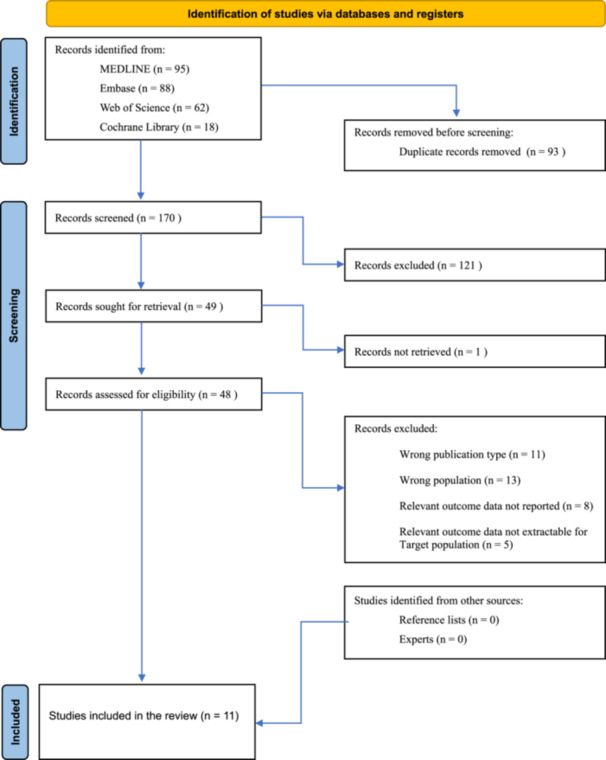
PRISMA flow diagram illustrating the study selection process. PRISMA, Preferred Reporting Items for Systematic Reviews and Meta‐Analyses.

The database searches identified a total of (*n* = 263) references, with no additional references identified in the grey literature. After deduplication, (*n* = 170) articles remained for title and abstract screening for title and abstract screening. (*n* = 121) references were excluded in the initial screening process, which left (*n* = 49) articles that were considered potentially relevant. One article was not retrieved, leaving (*n* = 48) whose full text was screened for eligibility. Following full text screening, (*n* = 37) additional references were excluded, and (*n* = 11) references that met the inclusion criteria were included [7, 9, 11, 19, 25, 26, 34, 36, 40, 43, 44]. Of the 37 studies excluded at full‐text level, reasons included: ineligible publication type (*n* = 11), ineligible study population (*n* = 13), relevant outcome data not reported (*n* = 8) or not extractable for the target population (*n* = 5). Figure 1 shows a PRISMA flowchart detailing the results of the literature search and review.

### Study characteristics

The characteristics of the included studies are shown in Table 2.

**Table 2:** Study characteristics and clinical outcomes of included studies.

Study		Country	No. rTKA	Strategy	Female (%)	Age in years (mean ± SD)	FU in years (mean ± SD, range)	Reinfection			Overall complications	Flap‐related complications	Functional outcomes
								1‐Year FU	2‐Year FU	Final FU			
Corten et al. [11]		Canada	24	Two‐stage	41.7	66.1 ± 9.3	4.4 ± 2.6 (1.0–10.0)	☓	☓	✓	✓	✓	✓
Tetreault et al. [40]		USA	21	Two‐stage	23.8	60.2 ± 13.4	3.5 ± 0.9 (2.0–5.9)	☓	☓	✓	✓	✓	✓
Warren et al. [43]		USA	25	Two‐stage	64.0	64.0 ± 10.4	3.5 ± 4.1 (0.1–18.0)	☓	☓	✓	✓	✓	☓
Cepas et al. [9]		Finland	20	One‐stage	b	66.8 ± 11.0	2.7 ± 2.5 (0.7–7.0)	☓	☓	✓	✓	✓	☓
Cepas et al. [9]		Finland	9	Two‐stage	b	66.8 ± 11.0	1.9 ± 1.3 (0.8–4.7)	☓	☓	✓	✓	✓	☓
Russo et al. [34]		Italy	15	Two‐stage	33.3	63.4 ± 12.5	5.4 ± 2.9 (2.0–10.0)	☓	☓	✓	✓	✓	✓
Sapino et al. [36]		CH	14	One‐stage	35.7	59.6 ± 9.9	2.7 ± 1.5 (0.6–5.3)	☓	☓	✓	✓	✓	☓
Sapino et al. [36]		CH	11	Two‐stage	63.6	72.2 ± 14.0	1.7 ± 1.2 (0.6–4.6)	☓	☓	✓	✓	✓	☓
Wiberg et al. [44]		Sweden	33	Two‐stage	64.0^a^	67.3 ± 11.2^a^	6.7 ± 3.4^a^	☓	☓	✓	✓	✓	☓
Kim et al. [19]		USA	33	Two‐stage	45.2	67.7 ± 10.0	3.5 ± 1.2 (2.0–6.8)	✓	☓	✓	✓	✓	☓
Müller et al. [26]		CH	1	One‐stage	100.0	85.0 (*n* = 1)	2.3 (*n* = 1)	✓	✓	✓	✓	✓	✓
Müller et al. [26]		CH	6	Two‐stage	50.0	75.2 ± 9.3	1.8 ± 0.7 (1.0–3.0)	✓	✓	✓	✓	✓	✓
McCulloch et al. [25]		UK	21	One‐stage	50.0	70.0 ± 19.8	4.4 ± 3.1 (0.3–10.7)	☓	☓	✓	☓	☓	☓
McCulloch et al. [25]		UK	2	Two‐stage	47.6	68.5 ± 7.9	4.5 ± 5.4 (0.7–8.3)	☓	☓	✓	☓	☓	☓
Brenneis et al. [7]		USA	38	Two‐stage	57.9	62.9 ± 15.4	2.5 ± 2.9 (0.4–9.4)	☓	☓	✓	✓	✓	☓

Abbreviations: FU, follow‐up; rTKA, revision total knee arthroplasty; SD, standard deviation; ✓, outcome reported in the study; *☓*, outcome not reported in the study.

^a^ Data refer to combined cohort; subgroup‐specific values are not reported separately.

^b^ Not reported.

### Risk of bias assessment

**Figure 2:**
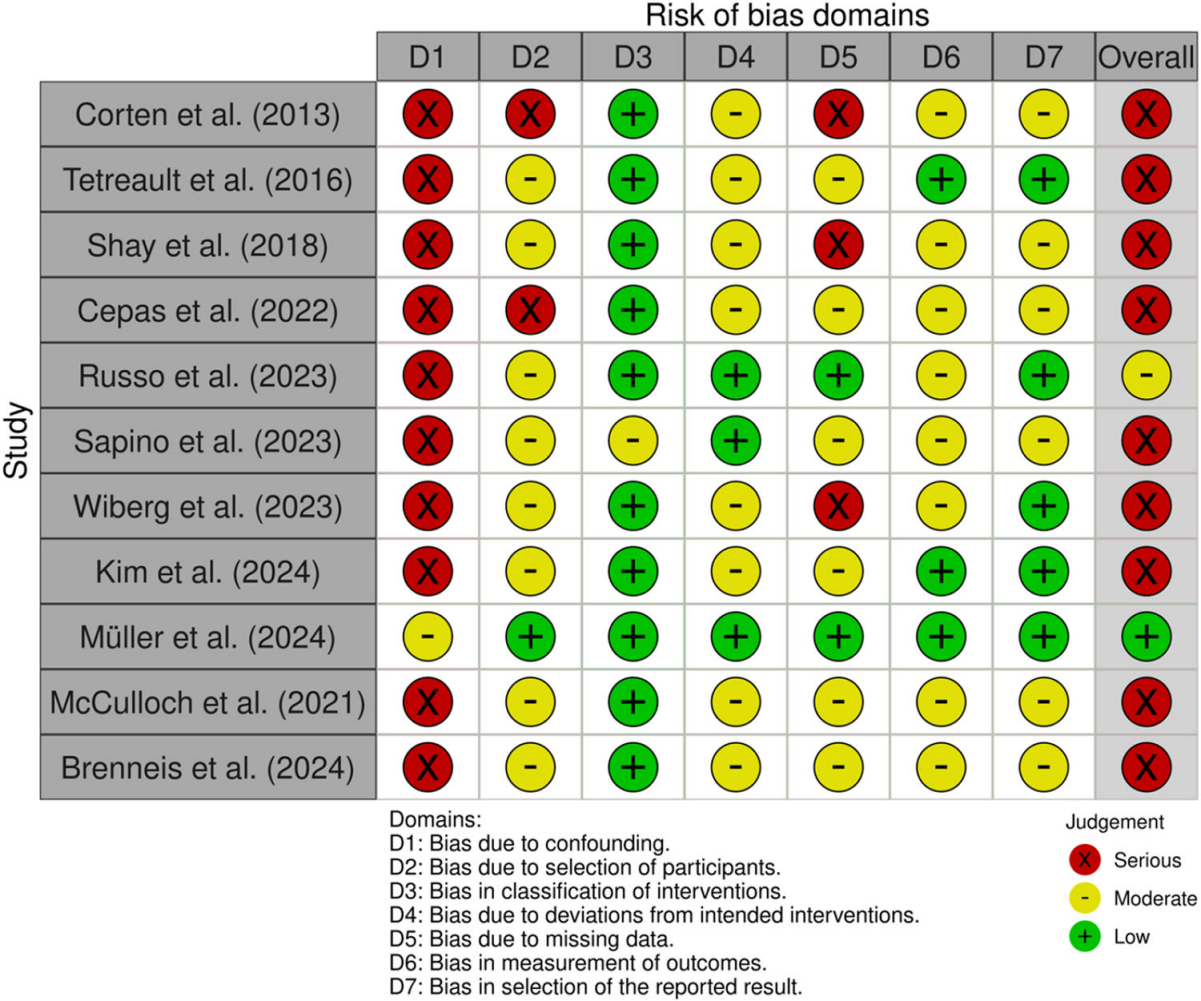
Traffic light plot displaying risk of bias per domain and overall judgement.

Risk of bias assessment is shown in Figure 2.

### Certainty of evidence assessment

Summary of the certainty of evidence is found in Table 3.

**Table 3:** GRADE evidence profile for reinfection, overall complications and flap‐related complications.

Certainty assessment						No. of patients		Effect		Certainty	Importance
No. of studies	Study design	Risk of bias	Inconsistency	Indirectness	Imprecision	One‐stage rTKA	Two‐stage rTKA	Relative (95% CI)	Absolute (95% CI)		
**Reinfection (follow‐up: mean 3.7 years; assessed with: Clinical, microbiological or radiographic evidence)**											
11	Non‐randomized studies	Serious^a^	Not serious	Not serious	Serious^b^	19/56 (33.9%)	98/215 (45.6%)	OR 0.61 (0.33–1.13)	118 fewer per 1000 (from 239 fewer to 30 more)	Low certainty (⊕⊕◯◯)^a^, ^b^	CRITICAL
**Overall complications (follow‐up: mean 3.7 years; assessed with: Surgical, medical, wound‐ or implant‐related events)**											
9	Non‐randomized studies	Serious^a^	Serious^c^	Serious^d^	Serious^e^	16/28 (57.1%)	84/184 (45.7%)	OR 1.59 (0.71–3.54)	115 more per 1000 (from 83 fewer to 292 more)	Very low certainty (⊕◯◯◯)^a^, ^c^, ^d^, ^e^	IMPORTANT
**Flap‐related complications (follow‐up: mean 3.7 years; assessed with: Complications directly related to the flap)**											
10	Non‐randomized studies	Serious^a^	Not serious	Serious	Not serious	8/28 (28.6%)	60/215 (27.9%)	OR 1.03 (0.43–2.47)	6 more per 1000 (from 136 fewer to 210 more)	Very low certainty (⊕◯◯◯)^a^	IMPORTANT

Abbreviations: CI, confidence interval; GRADE, Grading of Recommendations Assessment, Development and Evaluation; OR, odds ratio; ROBINS‐I, Risk Of Bias In Non‐randomized Studies—of Interventions.

^a^ Most included studies were retrospective cohort studies. According to ROBINS‐I, the majority were rated as having serious risk of bias due to confounding, participant selection and outcome measurement.

^b^ CIs include the possibility of both no effect and meaningful benefits, but not harm. Sample size and number of events were relatively small, resulting in limited precision.

^c^ Considerable heterogeneity in outcome reporting. Definitions, inclusion criteria for complications and methods of reporting varied substantially, limiting comparability and confidence in the pooled estimate.

^d^ Overall complications were not consistently or directly reported across studies. Reviewers derived complication rates by aggregating different event types and calculating rates, leading to indirectness in the evidence.

^e^ The CI around the effect estimate is wide and crosses the line of no effect, indicating uncertainty. Additionally, the small sample size in the one‐stage group limits precision and confidence in the estimate.

### Outcomes

#### PJIs

**Figure 3:**
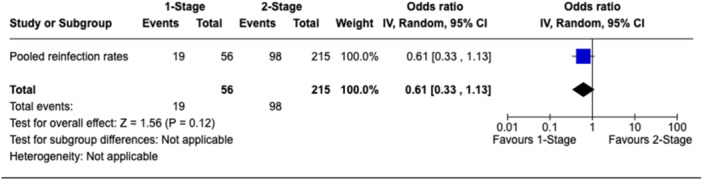
Forest plot comparing reinfection rates between one‐stage and two‐stage revision TKA with gastrocnemius flap coverage. CI, confidence interval; TKA, total knee arthroplasty.

A total of 11 studies [7, 9, 11, 19, 25, 26, 34, 36, 40, 43, 44] providing information on 271 rTKAs using a gastrocnemius flap reported reinfection rates. All 11 studies (100%, 271/271 rTKAs) reported reinfection rates at the final follow‐up, while only two studies reported information on 38 reinfection cases at 1‐ and 2‐year follow‐ups. Therefore, meta‐analysis was confined to data from the final follow‐up. Among the one‐stage group, PJI eradication at final follow‐up was achieved in 66.1% of cases (37 out of 56) at a mean follow‐up of 2.3 years (range, 2.0–2.7 years). In the two‐stage group, 54.4% (117 out of 215 cases) were infection‐free at the final follow‐up at a mean follow‐up of 3.2 years (range, 1.6–5.4 years). There was no statistically significant difference in PJI eradication rate between the one‐stage and two‐stage groups (OR: 0.61, 95% CI: 0.33–1.13, *p* = 0.12; Figure 3). Assessment of statistical heterogeneity was not applicable due to group‐level pooling.

#### Complications

**Figure 4:**
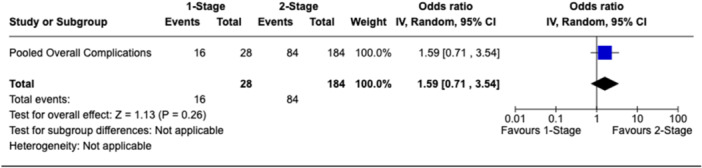
Forest plot comparing overall complication rates between one‐stage and two‐stage revision TKA with gastrocnemius flap coverage. CI, confidence interval; TKA, total knee arthroplasty.

Nine studies [7, 9, 11, 26, 34, 36, 40, 43, 44] (81.8%, 212 rTKAs [78.2%]) reported information related to the overall complication rate. Among the 212 knees with available data, 28 cases (13.2%) were treated with a one‐stage strategy, compared to 184 cases (86.8%) with a two‐stage strategy. The overall complication rate was similar in the one‐stage group (16/28 patients; 57.1%) compared to the two‐stage group (84/184; 45.7%) (OR: 1.59, 95% CI: 0.71–3.54, *p* = 0.26; Figure 4).

#### Flap‐related complications

**Figure 5:**
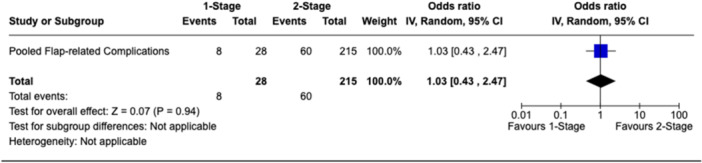
Forest plot comparing flap‐related complication rates between one‐stage and two‐stage revision TKA with gastrocnemius flap coverage. CI, confidence interval; TKA, total knee arthroplasty.

Ten studies [7, 9, 11, 19, 26, 34, 36, 40, 43, 44] with data on 243 rTKA cases reported on flap‐related complication rates. Of the 243 rTKA cases, 28 were treated with a one‐stage strategy (11.5%) and 215 with a two‐stage strategy (88.5%). The flap‐related complication rate was 28.6% (8/28) in the one‐stage group and 27.9% (60/215) in the two‐stage group, with no statistically significant difference in flap‐related complication rates between groups (OR: 1.03, 95% CI: 0.43–2.47, *p* = 0.94; Figure 5).

#### Functional outcomes

Functional outcomes were reported in four [11, 26, 34, 40] of the 11 included studies (36.4%), comprising 64 of 271 knees (23.6%) treated with gastrocnemius flap reconstruction. The KSS was reported in three studies [11, 34, 40] and the OKS in one study [34]. The ROM was reported in three studies [11, 26, 34] with flexion values ranging from 60° to 120°. Extension lag was reported in two studies [26, 34] but only for a subset of patients. Pain was not reported as a separate score in any study and could only be inferred from composite outcome measures such as the WOMAC or OKS. Additional scores included the WOMAC and SF‐12 each in a single study (Table 4). Due to substantial heterogeneity, no pooled analysis was performed.

**Table 4:** Summary of functional outcomes reporting and patient‐reported outcome measures of included studies.

Study	No. rTKA	Strategy	OKS (mean ± SD)	KSS (mean ± SD)	ROM (mean ± SD, range)	Extension lag (mean ± SD, range)	WOMAC (mean ± SD)	EQ‐5D (mean ± SD)	SF‐12 (mean ± SD)	
									Mental	Physical
Corten et al. [11]	24	Two‐stage	☓	103.0 ± NR	99.0 ± NR, 60°–110°	☓	51.0 ± NR	☓	49.76	32.17
Tetreault et al. [40]	21	Two‐stage	☓	☓	☓	☓	☓	☓	☓	☓
Warren et al. [43]	25	Two‐stage	☓	☓	☓	☓	☓	☓	☓	☓
Cepas et al. [9]	20	One‐stage	☓	☓	☓	☓	☓	☓	☓	☓
Cepas et al. [9]	9	Two‐stage	☓	☓	☓	☓	☓	☓	☓	☓
Russo et al. [34]	15	Two‐stage	36.0 ± 2.2	82.9 ± 4.8	83.0 ± NR, NR	7.5 ± 8.3, NR	☓	☓	☓	☓
Sapino et al. [36]	14	One‐stage	☓	☓	☓	☓	☓	☓	☓	☓
Sapino et al. [36]	11	Two‐stage	☓	☓	☓	☓	☓	☓	☓	☓
Wiberg et al. [44]	33	Two‐stage	☓	☓	☓	☓	☓	☓	☓	☓
Kim et al. [19]	33	Two‐stage	☓	☓	☓	☓	☓	☓	☓	☓
Müller et al. [26]	1	One‐stage	☓	☓	90.0 (*n* = 1)	15 ± (*n* = 1)	☓	☓	☓	☓
Müller et al. [26]	6	Two‐stage	☓	☓	106.0 ± 12.1, 90.0–120.0	5.8 ± 8.0 NR	☓	☓	☓	☓
McCulloch et al. [25]	21	One‐stage	☓	☓	☓	☓	☓	☓	☓	☓
McCulloch et al. [25]	2	Two‐stage	☓	☓	☓	☓	☓	☓	☓	☓
Brenneis et al. [7]	38	Two‐stage	☓	☓	☓	☓	☓	☓	☓	☓

Abbreviations: EQ‐5D, EuroQol‐5 Dimension; KSS, Knee Society Score; NR, specific data point not reported in the source publication (e.g., mean reported but standard deviation not provided); OKS, Oxford Knee Score; ROM, range of motion; rTKA, revision total knee arthroplasty; SF‐12, 12‐Item Short Form Health Survey; SD, standard deviation; WOMAC, Western Ontario and McMaster Universities Osteoarthritis Index; ☓, outcome measure not reported in the study.

## DISCUSSION

This review synthesized the evidence on rTKA with soft tissue reconstruction using a gastrocnemius flap for the treatment of PJI stratified by one‐stage and two‐stage approaches, with emphasis on reinfections, any complications and flap‐specific complications. Eleven studies provided data on 271 rTKAs. The most important finding of this systematic review and meta‐analysis was that with the data available in the literature, there was no significant difference in reinfection, any complication or flap‐specific complication rates between one‐ and two‐stage approaches.

Two‐stage revision has traditionally been considered the gold standard treatment and is still predominantly used [14]. However, the staged rTKA strategy is associated with higher surgical morbidity, longer hospitalization time, delayed functional recovery, psychological strain, high risk of complications during the interim period and increased cost [23, 38, 39]. As a result, interest in using a one‐stage approach has increased and is being used by more centres.

Several studies reported eradication rates following rTKA with gastrocnemius flap comparable to two‐stage revision. McCulloch et al. [25] observed a 57% rate in 21 patients undergoing single‐stage revision. Sapino et al. [36] treated 14 patients with a one‐stage and 11 with a two‐stage approach, both achieving 100% eradication. Cepas et al. [9] reported 50% eradication among 20 single‐stage cases and 44% in nine two‐stage cases. The findings of this review indicate that there was no statistically significant difference in reinfection or complication rates between one‐ and two‐stage procedures. This is in line with previous studies that have shown comparable reinfection rates for these interventions. However, there is likely to be clinical heterogeneity due to variations in factors such as type of infection (chronic/acute) and infectious agents as well as differences in follow‐up time.

The eradication rates observed in the present review (66.1% one‐stage, 54.4% two‐stage) are notably lower than benchmarks reported for revision TKA without flap reconstruction. Recent meta‐analyses report reinfection rates of 7.6%–12.7% for one‐stage and 8.8%–16.2% for two‐stage revision without flap coverage, corresponding to eradication rates of approximately 84%–92% [21, 41]. The lower success rates in the present cohort reflect the substantially greater complexity of patients requiring concomitant soft tissue reconstruction, who typically present with severe soft tissue compromise, extensive bone loss and higher infection burden.

This systematic review reveals a significant under‐reporting of functional outcomes. Only a small proportion of the studies analysed collected and reported information on validated PROMs and when they did, reporting was inconsistent. There is a considerable deficit in this area. For this vulnerable patient group in particular, quality of life and functional recovery are key concerns in limb salvage and central to patient‐centred decision‐making. Beyond functional outcomes, there was substantial under‐reporting of several clinically important variables likely to influence treatment success. Bacteriological characteristics, including causative microorganisms and antimicrobial susceptibility patterns, were inconsistently documented, precluding meaningful stratification by pathogen type or virulence. Antibiotic treatment strategies were similarly variably reported, with inconsistent information on agent selection, route and duration. Key surgical factors were also insufficiently described. Data on concomitant bone reconstruction, including the use of structural allografts, augments or custom implants and extensor mechanism repair were often not reported separately, and the type and constraint level of prosthetic implants were rarely specified in adequate detail. Collectively, these findings highlight important gaps in the existing literature. Inconsistent reporting of microbiological, surgical and treatment‐related variables limits the ability to perform clinically meaningful subgroup analyses and to identify patient‐ or procedure‐specific factors associated with treatment success or failure in this complex population. Future studies should therefore systematically incorporate PROMs alongside surgical and infection‐related outcomes to provide a holistic assessment of treatment outcomes.

One‐stage rTKA with a gastrocnemius flap may be a viable alternative to a two‐stage procedure for patients with PJI and a concomitant soft tissue defect, particularly when infection is early, mono‐microbial, bone volume is preserved and there is immediate access to permanent soft tissue coverage [16, 30, 45]. There are several advantages to a one‐stage strategy, including reduced cumulative surgical morbidity, a shorter overall treatment time, a shorter length of hospitalization and earlier functional recovery [12, 21, 41]. All of these factors may potentially benefit this vulnerable and exposed patient group. However, inadequate case selection and a lack of multidisciplinary management can lead to disastrous outcomes. Therefore, complex revision surgery involving flap coverage should only be performed in centres that perform revision arthroplasty and reconstructive soft tissue techniques frequently, ideally with a multidisciplinary team (MDT) involved.

This is the first comparative synthesis and the largest pooled dataset on this topic, conducted per PRISMA and assessed via ROBINS‐I and GRADE. To maximise sample size and reflect real‐world surgical heterogeneity, the review design was inclusive, incorporating all eligible studies reporting outcomes in rTKA with a gastrocnemius flap using either one‐ or two‐stage strategies. This approach was chosen as very few randomized comparative studies in the scientific literature on reconstructive and infection‐related arthroplasty. The comparability and quality of evidence in this heterogeneous area warrant improvement.

The study has a few limitations that should be acknowledged. It is worth noting that the present meta‐analysis includes the largest pooled cohort to date that compares both strategies in the context of rTKA with gastrocnemius flap coverage. However, interpretation is constrained by the small number of one‐stage cases, and most studies did not conduct direct comparative analyses. The total number of included studies was low and several included studies contributed small sample sizes, further reducing the precision of pooled estimates. Furthermore, the majority were retrospective. Based on the ROBINS‐I tool, all but two were deemed to be at serious risk of bias and the certainty of evidence was rated as low to very low across all outcomes using the GRADE framework. This limits the internal validity of the pooled results as the underlying data are methodologically weak. Secondly, follow‐up lengths varied widely between studies. This has the potential to introduce detection bias, especially with regard to late complications and reinfection. There was substantial clinical and methodological heterogeneity in surgical technique, flap timing, host factors, inconsistent definitions of infection chronicity and different settings for the indication of the respective surgical strategy, likely to have impacted the results. Hence, direct comparisons are difficult and the potential to make firm inferences from pooling the data is limited. It should also be noted that the analysis is based on study‐level rather than patient‐level aggregated data. This precludes further analysis of patient subgroups or outcome timing; for example, reinfection‐free survival. However, patient‐related factors could significantly impact outcomes. Further, relatively few cases were managed via a one‐stage rTKA. This led to wide CIs and limited statistical power for comparing surgical strategies. Lastly, the pooled outcome of the overall complications should be interpreted with caution. The definitions of complications varied considerably across studies. While some studies reported only major surgical events, others included medical or wound‐related complications without clearly defined criteria.

This review provides a comparative evidence synthesis to inform individualized treatment strategies for PJI with soft tissue defects. Future studies should harmonize outcome definitions and reporting, stratify by surgical approach, flap technique and infection characteristics and include validated functional outcomes with long‐term follow‐up. While high‐quality studies are challenging due to case rarity, patient heterogeneity and ethical constraints, coordinated multicentre registries could advance care for this high‐risk population.

## AUTHOR CONTRIBUTIONS

**Jonas Sina**: Conceptualization; formal analysis; investigation; data collection; visualization; writing—original draft; review and editing. **Daniel Schrednitzki**: Investigation; writing—review and editing. **Andrew Ting**: Investigation; data collection. **Daniel Addai**: Investigation; data collection. **Bassil Azam**: Investigation; data collection. **Priyanshu Saha**: Investigation; data collection. **Nils Meissner**: Writing; review and editing. **Andreas M. Halder**: Writing—review and editing. **Michael Dietrich**: Writing—review and editing. **Andrew James Price**: Writing—review and editing. **Abtin Alvand**: Supervision; conceptualization; writing—review and editing. **Alexander Maslaris**: Conceptualization; writing—review and editing.

## CONFLICT OF INTEREST STATEMENT

*Financial interests*: Jonas Sina has received funding from the Elite‐Med Foundation and the University of Oxford. Andreas M. Halder and Daniel Schrednitzki have received speaker and consultant honoraria from ZimmerBiomet. Andreas M. Halder and Nils Meissner have received research funding from the German Innovation Fund. Andreas M. Halder receives Royalties from ZimmerBiomet. Nils Meissner has received research funding from the German Society for Orthopaedics and Trauma and Aesculap AG. The remaining authors declare no conflict of interest. None of the funds received is relevant to this manuscript. *Non‐financial interests*: Andreas M. Halder is the President and Board Member of the European Knee Society. Andrew James Price is a Board Member of the European Knee Society.

## ETHICS STATEMENT

The authors have nothing to report.

## Supporting information

Supporting File

## Data Availability

The data are not publicly available due to privacy and ethical restrictions.
